# Determination of Prenatal Substance Exposure Using Meconium and Orbitrap Mass Spectrometry

**DOI:** 10.3390/toxics10020055

**Published:** 2022-01-26

**Authors:** Atakan Hernandez, Valerie Lacroze, Natalia Doudka, Jenny Becam, Carole Pourriere-Fabiani, Bruno Lacarelle, Caroline Solas, Nicolas Fabresse

**Affiliations:** 1 Laboratory of Pharmacokinetics and Toxicology, La Timone University Hospital, 264 Rue Saint Pierre, CEDEX 5, 13385 Marseille, France; atakan.hernandez@yahoo.fr (A.H.); jenny.becam@ap-hm.fr (J.B.); carole.pourriere-fabiani@ap-hm.fr (C.P.-F.); bruno.lacarelle@ap-hm.fr (B.L.); caroline.solas@ap-hm.fr (C.S.); 2 Department of Neonatalogy, La Conception University Hospital, CEDEX 5, 13385 Marseille, France; valerie.lacroze@ap-hm.fr; 3 Department of Clinical Pharmacology and Pharmacovigilance, La Timone University Hospital, 264 Rue Saint Pierre, CEDEX 5, 13385 Marseille, France; natalia.doudka@ap-hm.fr; 4 Emerging Viruses Unit (UVE), IRD 190, INSERM 1207, Aix-Marseille University, CEDEX 5, 13385 Marseille, France; 5 Economic and Social Sciences of Health and Medical Information Processing, INSERM, IRD, SESSTIM, Aix-Marseille University, CEDEX 5, 13385 Marseille, France

**Keywords:** meconium, mass spectrometry, toxicology, drug of abuse, orbitrap, newborn

## Abstract

The aim of this study was to develop and to validate a toxicological untargeted screening relying on LC-HRMS in meconium including the detection of the four main classes of drugs of abuse (DoA; amphetamines, cannabinoids, opioids and cocaine). The method was then applied to 29 real samples. Analyses were performed with a liquid chromatography system coupled to a benchtop Orbitrap operating in a data-dependent analysis. The sample amount was 300 mg of meconium extracted twice by solid phase extraction following two distinct procedures. Raw data were processed using the Compound Discoverer 3.2 software (Thermo). The method was evaluated and validated on 15 compounds (6-MAM, morphine, buprenorphine, norbuprenorphine, methadone, EDDP, amphetamine, MDA, MDMA, methamphetamine, cocaine, benzoylecgonine, THC, 11-OH-THC, THC-COOH). Limits of detection were between 0.5 and 5 pg/mg and limits of identification between 5 and 50 pg/mg. Mean matrix effect was between −79 and −19% (*n* = 6) and mean overall recovery between 18 and 73% (*n* = 6) at 100 pg/mg. The application allows the detection of 88 substances, including 47 pharmaceuticals and 15 pharmaceutical metabolites, cocaine and its metabolites, THC and its metabolites, and natural (morphine, codeine) and synthetic (methadone, buprenorphine, tramadol, norfentanyl) opioids. This method is now used routinely for toxicological screening in high-risk pregnancies

## 1. Introduction

In 2019, according to the United Nation Office on Drug and Crime (UNODC), cannabis remains the most widely used drug, with 200 million people having used cannabis, followed by opioids (62 million people), amphetamines (27 million people) and cocaine (20 million people) [1]. In France, 45% of adults (18–64 years old) have used cannabis, 5.6% cocaine, 5.0% 3,4-methylenedioxy-N-methylamphetamine (MDMA) and 1.3% heroin [2]. Drug of abuse (DoA) exposure during pregnancy may have serious consequences on newborn health: fetal development disorders, high neonatal mortality rates and various adverse mental and physical effects [3]. These concerns justify a recognition of fetal exposure as early as possible, in order to provide treatment to the exposed neonate. Maternal interview remains the most common and economical method to detect drug exposure, however, many studies have highlighted an underreporting issue [4]. Consequently, sensitive and specific bioanalytical methods are necessary to accurately measure biomarkers of in utero exposure. Fetal drug exposure can be identified by analyzing maternal specimens during pregnancy or neonatal specimens such as hair, urine and meconium, shortly after birth.

Meconium refers to the first stool of the newborn. Meconium consists mainly of water, epithelial cells, lanugo, bile acids and salts, cholesterol and sterol precursors, blood group of substances, mucopolysaccharides, sugars, lipids, proteins and other compounds from swallowed amniotic fluid [5]. It accumulates during the last three months of pregnancy, thus allowing exploration of the exposure of the newborn during the last trimester of pregnancy.

Meconium is accepted as a gold standard matrix for in utero drug exposure. It allows for a wide detection window and a non-invasive sample collection from a soiled diaper. However, it is susceptible to contamination by urine, there is a possible detection of drugs given to the newborn after birth and painkiller drugs used by the mother during labor, the specimen volume is limited and extensive extraction procedures are required for sample preparation [6]. Several studies dedicated to meconium analysis have been published [7,8,9,10,11,12,13,14,15,16,17,18,19,20]. Most of the methods have focused on the analysis of specific drug groups such as amphetamines, opioids, cannabinoids, cocaine, alcohol biomarkers, benzodiazepines or antidepressants [7,8,9,10,11,12,13,14,15,16]. Typically, each drug group is extracted and analyzed separately in meconium; this may cause problems due to the limited amount of sample, and the time-consuming procedures required for multiple drugs analysis. Very few methods devoted to large screening of meconium have been published. The first broad-spectrum drug screening of meconium was developed by Ristimaa et al. in 2010 [17]. This method relies on liquid chromatography coupled to high resolution mass spectrometry (LC-HRMS) and an in-house database containing 869 molecular drugs. More recently (2021), López-Rabuñal et al. proposed another large screening devoted to new psychoactive substances (NPS) [21]. This method allows the simultaneous determination of 137 NPS in meconium by LC-HRMS. These two methods are efficient and innovative; however, they are targeted. To our knowledge, there is no untargeted LC-HRMS method devoted to toxicological screening in meconium, and none allowing the simultaneous detection of the four main classes of DoA (amphetamines, cannabinoids, opioids and cocaine) in the current literature.

The aim of this study was to develop and to validate a toxicological untargeted screening relying on high resolution mass spectrometry (Orbitrap) in meconium, including the detection of the four main classes of DoA. The method was then applied to real samples.

## 2. Materials and Methods

### 2.1. Chemicals and Reagents

Water purity was 18.2 mΩ/cm (Millipore, Molsheim, France). Methanol was supplied by Biosolve (Dieuze, France). Formic acid and orthophosphoric acid were ordered to Carlo Erba (Val de Reuil, France). Acetonitrile and sodium dihydrogen phosphate (NaH_2_PO_4_) were supplied by Fisher Scientific (Illkirch, France). Sodium hydrogen phosphate (Na_2_HPO_4_) was ordered from Merck (Darmstadt, Germany). β-glucuronidase was purchased by MP Biomedicals (Illkirch, France). LGC standards (Molsheim, France) supplied vials for 11-hydroxy-Δ^9^-tetrahydrocannabinol (11-OH-THC), 11-nor-9-carboxy-Δ^9^-tetrahydrocannabinol-D3 (THC-COOH-D3), Δ^9^-tetrahydrocannabinol-D3 (THC-D3), methamphetamine, cocaine, 3,4-methylenedioxyamphetamine (MDA), 3,4-methylenedioxymetamphetamine (MDMA), MDMA-D5, morphine-D3, buprenorphine, methadone, 6-monoacetylmorphine (6-MAM), 6-MAM-D3. Vials of 11-OH-THC, THC-COOH, THC, methampethamine-D5, amphetamine, amphetamine-D5, cocaine, cocaine-D3, MDA-D5, benzoylecgonine, benzoylecgonine-D3, morphine, buprenorphine-D4 and methadone-D3 were supplied by Euromedex (Souffelweyersheim, France).

### 2.2. Solutions Preparation

Phosphate buffer pH = 5 was made with 1.70 g Na2HPO4 and 12.14 g NaH2PO4 in 1000 mL of distilled water. The pH was adjusted with orthophosphoric acid. The elution phase was prepared with 90 mL acetonitrile and 10 mL methanol. A stock solution at 10 µg/mL was prepared in methanol by mixing all the standards with appropriates volumes. The internal standard solution was a mixture of all the deuterated compounds at 5 µg/mL in methanol.

### 2.3. Sample Preparation

**Figure 1:**
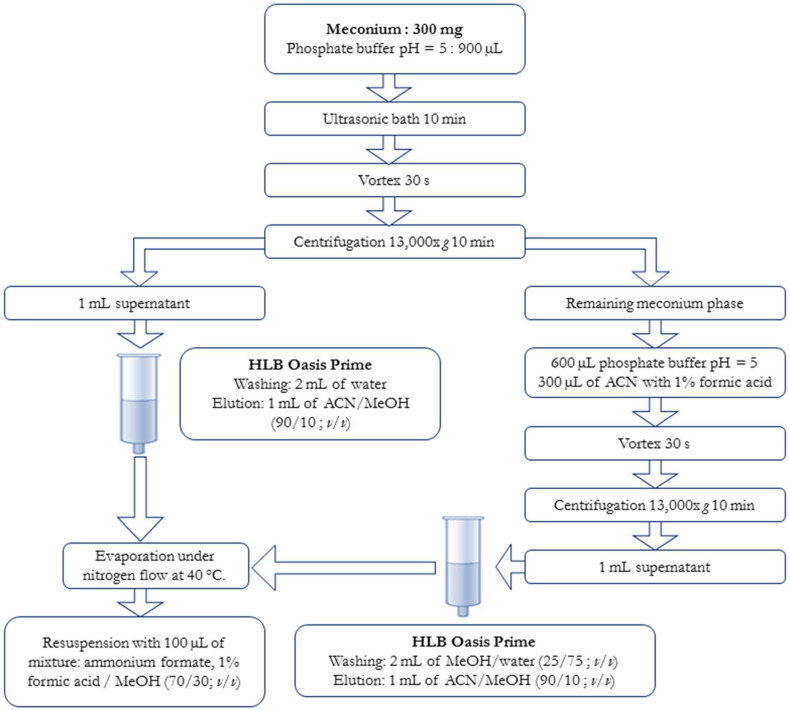
Schematic representation of the extraction procedure.

Meconium samples are stored at −80 °C before analysis. Two solid phase extractions (SPE) are carried out from the sample: (1) a first extraction devoted to DoA non cannabinoids, and (2) a second one devoted to cannabinoids. The sample amount is 300 mg of meconium (±10 mg) and 900 µL of phosphate buffer pH = 5 were added. The mixture is vortexed for 20 s following by 10 min in an ultrasonic bath. The mixture is then hydrolyzed with 100 µL of β-glucuronidase at 48 °C for 1 h. A first centrifugation is done, 10 min at rpm. (1) The supernatant is transferred into an Oasis HLB Prime column (Waters, Milford, CT, USA), which were not previously conditioned. The column is rinsed with 2 mL of water and dried for 10 min under vacuum. The molecules are then eluted with 1 mL of an acetonitrile/methanol mixture (90/10; *v/v*). (2) Residual meconium is reconstituted with 600 µL of buffer pH = 5 and 300 µL of acetonitrile with 1% formic acid, the sample is vortexed for 20 s and then centrifuged for 10 min at rpm. The supernatant is transferred into a novel Oasis HLB Prime column without pretreatment. After introducing the sample, the column is rinsed by adding 2 mL of methanol/water (25/75; *v/v*) and dried for 10 min under vacuum. The molecules are then eluted with 1 mL of an acetonitrile/methanol (90/10; *v/v*). The eluates are collected before being evaporated to dryness under nitrogen flow at +48 °C. The dry residue is reconstituted with 100 µL of mobile phase A (2 mM ammonium formate in water and 0.1% formic acid)/methanol (70/30; *v/v*). Five µL is injected into the chromatographic system. A schematic description of the procedure is presented in Figure 1.

### 2.4. Instruments

#### 2.4.1. Liquid Chromatography

Liquid chromatography was performed on a Vanquish UHPLC system (Thermo, Les Ulis, France). The compounds were separated on a Luna Omega Polar C18 column (2.1 mm × 100 mm; 1.6 µm, Phenomenex, Le Pecq, France) with an oven temperature set at 50 °C. The flow rate was fixed at 0.4 mL/min. The mobile phase A was a mixture of 2 mM ammonium formate in water and 0.1% formic acid; mobile phase B was methanol. The chromatographic gradient was as follows: 100% A for 1 min, linear gradient to 55% A in 5 min, linear gradient to 50% A in 1 min held for 1 min, follow by a linear gradient to 0% A in 4 min held for 1 min. The column re-equilibration was performed by a linear gradient to 100% A in 0.1 min held for 2 min.

#### 2.4.2. Mass Spectrometry

Ionization was performed with a heated electrospray ionization (HESI) source operating in positive mode. Nitrogen was employed as sheath gas (60 UA) and auxiliary gas (10 UA). Vaporization temperature was set at 320 °C. Capillary voltage was set at 3.5 kV and S-lens at 70 eV. The ions were then analyzed by high-resolution mass spectrometry (Orbitrap Exploris 120, Thermo, Les Ulis, France) in data-dependent mode. The full scan analysis was realized over a window ranging from 125 to 650 *m*/*z* with a resolution of 60,000 FWHM. The MS2 analysis was performed with a parent ion isolation window of 1 *m*/*z*, a dynamic exclusion of 6 s and a resolution of 16,000 FWHM. Mass spectrometer was calibrated once a week with a MRFA solution (L-methionyl-arginyl-phenylalanyl-alanine acetate) 1 µg mL^−1^, caffeine 2 µg mL^−1^ and Ultramark^®^ 1621 0.001% over a mass range of 50–2000 *m*/*z*.

### 2.5. Data Reprocessing

Data were processed using the Compound Discoverer 3.2 (Thermo, Les Ulis, France) software following a specific workflow. All ions presenting a signal over 3 times the background noise and a peak intensity over 500,000 were taken into account to create the extracted ion chromatogram (EIC). MS and MS2 spectra were then used to identify ions. Three processes were used for compound identification:

(1) MassList: this process includes an in-house library containing 150 molecules. Identification is carried out using exact mass, isotopic profile and retention time.
(2) MzCloud: mzCloud™ is an online library containing 19,521 molecules with MS and MS2 spectra [22]. The mzCloud™ database contains 17 compound classes, screening was performed including all classes. Identification was performed using the HighChem HighRes algorithm.
(3) NIST: the NIST is a downloaded library constituted by the National Institute of Standards and Technology, recently a spectra database compatible with LC-HRMS technology has been released. This library contained 26,000 molecules with MS and MS2 spectra. The identification is performed using the NIST identification algorithm.

These three processes include monoisotopic mass and isotopic pattern for compound identification with a tolerance of 5 ppm.

### 2.6. Method Validation

The method was validated for 15 molecules: 6-MAM, morphine, buprenorphine, norbuprenorphine, methadone, EDDP, methamphetamine, amphetamine, MDA, MDMA, cocaine, benzoylecgonine, THC, 11-OH-THC and THC-COOH. SWGTOX guidelines were followed for the validation procedure [23]. The following criteria were considered for a qualitative method validation: specificity, limit of detection, limit of identification, matrix effect, extraction yield and cross-contamination (carry over).

#### 2.6.1. Specificity

Specificity was assessed by analyzing five drug-free meconium samples from healthy newborns. Currently, no commercial proficiency test is available for meconium toxicological screening.

#### 2.6.2. Matrix Effect and Extraction Yield

Three procedures (A, B and C) were performed on six different blank meconium samples at two concentrations (100 pg/mg and 500 pg/mg) in order to evaluate extraction yield and matrix effect (ME): (A) Analytes and the IS were spiked in the mobile phase and directly injected; (B) Analytes and the IS were spiked afterwards in extracted blank matrix samples and injected; and (C) Analytes and IS were spiked in meconium samples, the complete extraction procedure was carried through, and the samples were injected into the system. The mean chromatographic peaks obtained using the three procedures were compared. The ratios C/B, B/A and C/A determined the extraction yield, the matrix effect and the process efficiency, respectively, and were calculated for each analyte.

#### 2.6.3. Limit of Detection and Identification

Sensitivity was evaluated by injecting 3 different meconium matrices spiked with a mixture of the 15 substances at different concentrations (0.05 pg/mg, 0.1 pg/mg, 0.5 pg/mg, 1 pg/mg, 5 pg/mg, 10 pg/mg, 50 pg/mg, and 100 pg/mg). Limit of identification (LOI) was defined by the lowest concentration of analyte that could be correctly identified by the processing software. Limit of detection (LOD) was defined as the lower concentration exhibiting a signal at least three-fold the background noise.

#### 2.6.4. Cross-Contamination

Cross-contamination was assessed by injecting a blank sample immediately after a blank sample spiked at 500 pg/mg (50 pg/mg for 6-MAM, buprenorphine, norbuprenorphine, THC, THC-COOH and 11-OH-THC). For a quantitative method, the signal generated must be lower than 20% of the limit of quantification for the analytes and 5% for the internal standards. In the development of this qualitative method, we consider that no signal should be generated on the blank sample.

#### 2.6.5. Application

The method was applied to real samples addressed to the Pharmacokinetics and Toxicology Laboratory of Marseille. Meconium samples were collected between 0 and 3 days after birth, and sent to the laboratory at ambient temperature. Samples were then stored at −80 °C till analysis. Stability after 3 freeze/thaw cycles was evaluated in previous studies. No relevant degradation was observed for 6-MAM, morphine, cocaine, benzoylecgonine, buprenorphine, norbuprenorphine, THC, THC-COOH and 11-OH-THC (<10%), for amphetamine, metamphetamine, MDA, MDMA (<15%) and for methadone and EDDP (<20%) [7,24,25,26,27,28,29,30].

## 3. Results and Discussion

### 3.1. Method Development and Validation

The aim of this study was to develop a new analytical method devoted to meconium toxicological screening relying on Orbitrap mass spectrometry. Meconium remains the gold standard matrix to evaluate in utero exposure to xenobiotics, however two major limits reduce its use. The matrix is very pasty and sticky, requiring a complex analytical pre-treatment and the sample amount is sometimes limited, allowing a single analysis. In the method presented here, a small amount of sample was necessary, 300 mg, which is acceptable in comparison to previous publications using between 200 and 2000 mg [7,8,9,10,11,12,13,14,15,16,17,18]. Several extraction procedures have been applied to meconium toxicological analysis including liquid–liquid extraction [9], salting out assisted liquid–liquid extraction [14] and solid-phase extraction (SPE) [11,12,13,15,16,17,18]. SPE is widely employed to prepare and clean up complex matrices in the field of forensic analysis and is the most widely used method for the preparation of meconium. SPE was therefore chosen in this method development. The chromatographic run was completed in 12 min and initial conditions were restored in 2 min. No interferences were observed for the 15 compounds included in the method evaluation after the analysis of 5 blank meconium samples.

Mean matrix effects and extraction recoveries results are presented in Table 1. An ion suppression was observed for all compounds between −79 and −19% at 100 pg/mg and −89% and −16% at 500 pg/mg. These modifications of ionization were well corrected with internal standards, providing ME between −15 and +15% with the exception of 6-MAM at 100 pg/mg (+28%). Coefficients of variation were under 15%, highlighting a good precision, although this method is not devoted to quantitation. Cannabinoids (THC, 11-OH-THC and THC-COOH) presented the most important ion suppression (between −89 and −49%). Prego-Meleiro et al. noticed a similar matrix effect in meconium (between −71 and −26%) [16]. However, this does not affect the sensitivity with a limit of detection for cannabinoids at 5 pg/mg and a limit of identification at 10 pg/mg (with the exception of THC-COOH, not identified). Extraction recoveries were over 50% for most compounds except norbuprenorphine at 500 pg/mg (47%) and cannabinoids (between 18 and 45%). Ristimaa et al. obtained a lower extraction recovery (16%) for THC-COOH after liquid–liquid extraction [17]. Prego-Meleiro et al. observed a higher extraction yield, between 50 and 68%, however their sample pretreatment relying on a SPE was specifically devoted to the detection and quantification of cannabinoids and their metabolites in meconium [16].

Limits of detection and identification are presented in Table 2. The LOD are in good agreement with previously published methods. LOD obtained for 6-MAM, EDDP, metamphetamine, amphetamine, MDMA, cocaine and THC are slightly higher than those reported in the literature. This could be attributed to the mass spectrometer acquisition mode. The detection is carried out from the spectrum acquired in fullscan mode, which is more affected by the background noise than data acquired following a LC-MS/MS acquisition (MRM) used in the majority of the methods indicated in Table 2. A comparison of LOD and meconium concentrations measured in real samples is also provided in Table 2. The developed method is sensitive enough to detect all compounds. The only compound exhibiting a LOD overlapping meconium concentrations is THC: LOD = 5 pg/mg and concentrations measured in real samples = 4.2–7.7 pg/mg. However, THC is always detected in association with at least one metabolite, and exhibits concentration lower than THC-COOH in meconium samples [16]. Therefore, this limit is easily compensated for by the detection of THC-COOH. To our knowledge, limits of identification have never been evaluated before in meconium for these compounds with high resolution mass spectrometry and an untargeted approach (no inclusion list). All molecules have been successfully identified at low concentrations (<50 pg/mg) with the exception of THC-COOH. Interestingly, LOI were lower enough to identify most compounds at the concentrations found in real samples. The only class requiring a targeted approach remains cannabinoids. Regarding carry-over, blank samples injected after meconium samples spiked at 500 pg/mg did not present any traces for the 15 validated molecules.

### 3.2. Application

**Figure 2:**
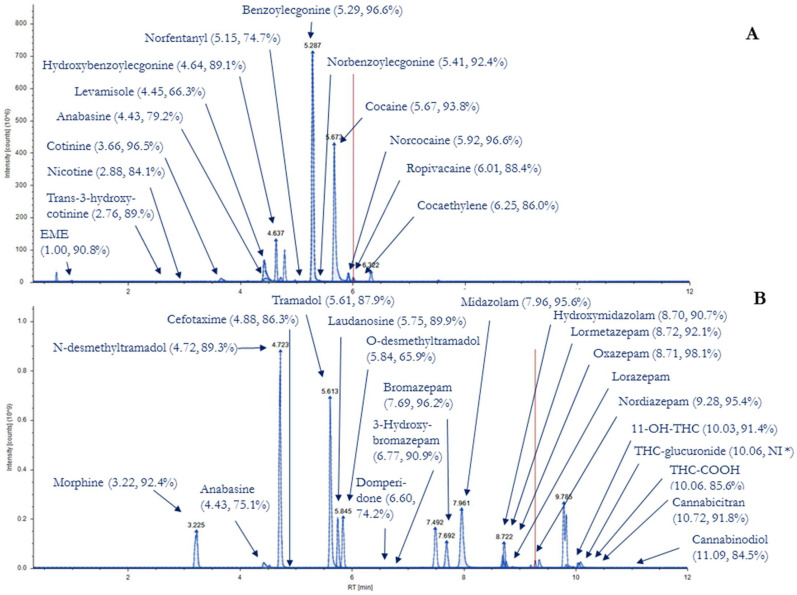
Presentation of the extracted ion chromatograms (EIC) for the substances identified in sample nº 4 (**A**) and sample nº 14 (**B**) (retention time, similarity score with the MzCloud™ spectral library). * THC-glucuronide was not identified with MzCloud™ and NIST library (NI), but solely with the house made library.

The results of meconium analyses (*n* = 29 samples) are presented in Table S1. The application allowed the identification of different biomarkers in meconium samples. All molecules included in the validation were detected with the exception of amphetamines (amphetamine, metamphetamine, MDMA and MDA) and 6-MAM. However, these molecules were included in the method validation, and presented LOD lower than the concentrations observed in the literature. These molecules were therefore probably absent from the samples analyzed. THC-COOH (not identified during LOI evaluation) was successfully identified in 3 samples; the THC-COOH LOI probably corresponds to a high concentration, higher than those evaluated in the method validation. EIC of the molecules identified in samples 4 and 14 are presented in Figure 2.

The untargeted screening allowed the detection of 88 different substances, each substance being detected in 1 to 22 samples. They include 47 pharmaceuticals and 15 pharmaceutical metabolites (antalgic, antibiotics, anticonvulsants, antidepressants, antiemetic, anti-histaminics, antihypertensives, antipyretic, antiretrovirals, benzodiazepines, beta-2-agonist, beta-blocker, H2 blocker, local anesthetics, antifungal, neuroleptics, opiates, proton pump inhibitor, and stimulant). Most of these molecules are classically prescribed to pregnant woman for nausea, gastroesophageal reflux, peripartum anesthesia, infection prevention during cesarean section and eclampsia treatment.

Cocaine and its metabolites were successfully identified (benzoylecgonine (*n* = 5), cocaethylene (*n* = 1), cocaine (*n* = 4), ecgonine methyl ester (*n* = 2), hydroxybenzoylecgonine (*n* = 1), norbenzoylecgonine (*n* = 2), norcocaine (*n* = 1)). These substances are commonly identified in meconium [7,28,34]. The identification of cocaethylene in one sample is of particular importance since this metabolite allows the identification of fetal alcohol exposure in addition to cocaine. Levamisole, a cocaine adulterant, was detected in one sample. To our knowledge, levamisole is identified for the first time in meconium. Cannabinoids were identified in 10 samples (11-OH-THC (*n* = 5), cannabicitran (*n* = 1), cannabidiol (*n* = 1), cannabinol (*n* = 8), THC (*n* = 4), THC-COOH (*n* = 7), THC-COOH-glucuronide (*n* = 7)). This class is of particular importance since this is the main DoA used in France and especially in the Marseille region [2,35]. Natural opioids (codeine (*n* = 2), norcodeine (*n* = 1), morphine (*n* = 5), morphine-3-glucuronide (*n* = 1), normorphine (*n* = 1)), synthetic opioids (tramadol (*n* = 3), N-desmethyltramadol (*n* = 2), O-desmethyltramadol (*n* = 1), norfentanyl (*n* = 16)) and opioid substitution treatments (methadone (*n* = 2), EDDP (*n* = 2), buprenorphine (*n* = 1), norbuprenorphine (*n* = 1)) were well detected. Interestingly, solely norfentanyl (*n* = 16) was identified and fentanyl was not detected even following a manual identification. Conversely, López-Rabuñal et al. identified fentanyl in four meconium samples without norfentanyl (included in the screening) [21]. Ristimaa et al. detected fentanyl in 2 out of 209 meconium samples, however they do not indicate if norfentanyl was screened for [17]. Fentanyl is known to cross the placenta barrier and has been detected in umbilical cord plasma [36]. Therefore, this compound should be detected in meconium samples. This discrepancy could be explained by a lack of sensitivity, as fentanyl LOD and LOI were not evaluated here. Additionally, several biomarkers of tobacco exposure were identified (anabasine (*n* = 22), nicotine (*n* = 3), cotinine (*n* = 20), cotinine-N-oxide (*n* = 3), trans-3-hydroxycotinine (*n* = 4)).

Most of the analytical methods described in the literature for the determination of DoA in meconium are based on LC-MS/MS [10,11,12,13,14,15,16,18]. All these methods are targeted, therefore limiting the number of compounds analyzed. LC-HRMS allows the inclusion of hundreds of compounds in the database without compromising on sensitivity. In addition, retrieval of new compounds in the acquisition data is easy because the formula database is updatable with literature data for current substances, such as designer drugs. These results highlight the interest of this untargeted analytical method in the context of high-risk pregnancies. The application to samples collected in the framework of the care of newborns has made it possible to highlight most of the molecules of interest. This made it possible to document in a precise and almost exhaustive manner the exposure of newborns in utero, to adapt medical monitoring and care at birth and in particular to adjust treatment in the event of a withdrawal syndrome. The main limitation of this method is the absence of biomarker of exposure to alcohol. The qualitative aspect of the developed method may be a second limitation; however, interpretation of quantitative results is difficult due to contamination of meconium with urine [37,38,39]. A further limitation of this procedure is the cumbersome analytical preparation prior the analysis (two SPE). However, meconium is a tricky matrix which requires an extensive analytical pre-treatment.

## 4. Conclusions

This study enabled the development of a sensitive untargeted method devoted to meconium analysis, using low amount of meconium. To our knowledge, this is the first untargeted method allowing the simultaneous detection of the four categories of DoA (opioids, amphetamines, cannabinoids, cocaine). The application to real samples demonstrates the efficacy of this protocol in identifying DoA and pharmaceuticals. This method is now used routinely for toxicological screening in high-risk pregnancies. This procedure, by revealing the presence of drugs and metabolites beyond the ordinary scope of abused drugs, will significantly help pediatricians and will make it possible to quickly adapt the care of newborns.

## Figures and Tables

**Table 1:** Matrix effect and coefficient of variation observed for the 15 compounds at 100 pg/mg and 500 pg/mg (compounds marked with an asterisk (*) were evaluated at 10 pg/mg and 50 pg/mg).

	100 pg/mg (*n* = 6)					500 pg/mg (*n* = 6)				
Compounds	Matrix Effect			Extraction Yield (%)	Process Efficiency (%)	Matrix Effect			ExtractionYield (%)	Process Efficiency (%)
	Raw (%)	Normalized with Internal Standard (%)	CV (%)			Raw (%)	Normalized with Internal Standard (%)	CV (%)		
6-MAM *	−29	+28	10.8	52	37	−29	+15	14.9	53	38
Morphine	−42	+4	9.6	61	35	−31	+11	10.7	55	38
Buprenorphine *	−25	−3	5.3	66	50	−27	+11	10.5	61	45
Norbuprenorphine *	−31	−4	9.1	62	43	−39	+4	11.7	47	29
Methadone	−19	0	10.2	70	57	−18	+13	11.6	61	50
EDDP	−22	−3	5.8	71	55	−19	+11	11.2	72	58
Amphetamine	−35	−1	12	73	47	−22	+23	12.2	61	48
MDA	−29	−1	9.6	61	43	−16	+15	12.2	68	57
MDMA	−31	−3	6.7	60	41	−25	+16	9.3	62	47
Methamphetamine	−31	−5	7.4	69	48	−23	+13	9.7	65	50
Cocaine	−25	−5	6.1	61	46	−21	+11	12.7	64	51
Benzoylecgonine	−25	−2	7.6	62	47	−26	+10	10.9	63	47
THC *	−79	−2	8.2	24	5	−89	−12	14.9	19	2
11-OH-THC *	−49	+5	10.1	45	23	−65	+6	8.3	31	11
THC-COOH *	−69	+14	11.6	18	6	−72	+10	14.4	18	5

**Table 2:** Limit of detection (LOD) and limit of identification (LOI) observed for the 15 compounds, LOD retrieved in previous studies and concentrations.

Compounds	LOD (pg/mg)	LOI (pg/mg)	LOD Found in the Literature (pg/mg)	Concentrations Found in the Literature (pg/mg)	References
6-MAM	5	5	0.3–1.5	5–142 (*n* = 3)	[7,17,28]
Morphine	0.5	10	1.2–6	397 (*n* = 1)	[7,17,28]
Buprenorphine	0.5	5	5–10	23.9–240.5 (*n* = 9)	[17,31,32]
Norbuprenorphine	5	10	5–10	323.9–1880.2 (*n* = 10)	[17,31,32]
Methadone	0.1	5	0.25–10	85–21,980 (*n* = 48)	[17,28,33]
EDDP	0.5	5	0.25–25	4431–101,021 (*n* = 48)	[28,33]
Methamphetamine	1	50	0.2–10	18–13,325 (*n* = 16)	[14,17,27]
Amphetamine	5	5	0.5–10	41–2220 (*n* = 15)	[14,17,27]
MDA	5	5	2–4	No data	[14,17]
MDMA	5	10	0.3–4	No data	[14,17]
Cocaine	1	50	0.5–0.9	72–903 (*n* = 3)	[7,28]
Benzoylecgonine	0.5	5	1–1.2	134–847 (*n* = 3)	[7,28]
THC	5	10	1	4.2–7.7 (*n* = 4)	[16]
11-OH-THC	5	10	1	11.9 (*n* = 1)	[16]
THC-COOH	5	NI	1–20	24.1–288.8 (*n* = 4)	[16,17]

## Data Availability

Not applicable.

## Supplementary Materials

The following are available online at https://www.mdpi.com/article/10.3390/toxics10020055/s1, Table S1: Substances identified in meconium samples.
