# Supplementary material for: Determination of Prenatal Substance Exposure Using Meconium and Orbitrap Mass Spectrometry
Source: Toxics. 2022 Jan 26;10(2):55. doi: 10.3390/toxics10020055 (PMC8875502; doi:10.3390/toxics10020055)
Supplement: Supplementary file 1 [file toxics-10-00055-s001.zip › toxics-1546633-supplementary.pdf]

toxics

MDPI

Supplementary Materials: Determination of prenatal substance exposure using meconium and Orbitrap mass spectrometry

Atakan Hernandez, Valerie Lacroze, Natalia Doudka, Jenny Becam, Carole Pourriere-Fabiani, Bruno Lacarelle, Caroline Solas and Nicolas Fabresse

Table S1. Substances identified in meconium samples.

N°.	Substances identified in meconium
1	**Biomarkers of tobacco exposure:**<br>Anabasine, nicotine, cotinine-N-oxide, trans-3-hydroxycotinine, cotinine<br>**Biomarkers of cannabis exposure:**<br>Cannabinol, THC-COOH-glucuronide
2	**Biomarkers of tobacco exposure:**<br>Anabasine, cotinine<br>**Biomarkers of cannabis exposure:**<br>Cannabinol; THC-COOH, THC-COOH-glucuronide<br>**Pharmaceuticals:**<br>Ropivacaine, lidocaine, prilocaine<br>clobazam, diazepam, nordiazepam, 4-hydroxynordiazepam, oxazepam, temazepam, norfentanyl, salbutamol
3	**Biomarkers of tobacco exposure:**<br>Anabasine, cotinine, trans-3-hydroxycotinine<br>**Biomarkers of cannabis exposure:**<br>Cannabinol, THC-COOH-glucuronide<br>**Biomarkers of cocaine exposure:**<br>Cocaine, benzoylecgonine<br>**Pharmaceuticals:**<br>Metoclopramide, norfentanyl, pantoprazole, paracétamol, ropivacaine, lidocaine
4	**Biomarkers of tobacco exposure:**<br>Anabasine, nicotine, cotinine, trans-3-hydroxycotinine<br>**Biomarkers of cocaine exposure:**<br>Cocaine, norcocaine, benzoylecgonine, ecgonine methyl ester, norbenzoylecgonine, levamisole (cocaine adulterant), cocaethylene (biomarker of alcohol and cocaine coexposure)<br>**Pharmaceuticals:**<br>Norfentanyl, ropivacaine
5*	**Biomarkers of tobacco exposure:**<br>Anabasine, cotinine, nicotine, cotinine-N-oxide, trans-3-hydroxycotinine<br>**Biomarkers of cannabis exposure:**<br>Cannabinol, THC-COOH, THC-COOH-glucuronide<br>**Biomarkers of cocaine exposure:**<br>Cocaïne, benzoylecgonine<br>**Pharmaceuticals:**<br>Lidocaine, 3-hydroxylidocaine, ropivacaine, norfentanyl, quétiapine
6	**Biomarkers of tobacco exposure:**<br>Anabasine, cotinine<br>**Biomarkers of cannabis exposure:**<br>11-OH-THC, cannabinol, THC-COOH-glucuronide<br>**Pharmaceuticals:**<br>Lidocaine, norfentanyl, ropivacaine, secnidazole
7	**Biomarkers of tobacco exposure:**<br>Anabasine, cotinine, cotinine-N-oxide<br>**Biomarkers of cocaïne exposure:**

	Benzoylecgonine, hydroxybenzoylecgonine, norbenzoylecgonine<br>**Pharmaceuticals:**<br>Methadone, EDDP, lidocaine
**8**	**Biomarkers of tobacco exposure:**<br>Cotinine<br>**Pharmaceuticals:**<br>Buprenorphine, norbuprenorphine, norfentanyl, diazepam, oxazepam, quetiapine
**9**	**Pharmaceuticals:**<br>Bupivacaine, cefazoline, cimetidine, cyamemazine, ropivacaine
**10**	**Biomarkers of tobacco exposure:**<br>Cotinine, anabasine<br>**Pharmaceuticals:**<br>Oxazepam, norfentanyl
**11**	NF
**12**	**Biomarkers of cannabis exposure:**<br>THC, 11-OH-THC
**13**	**Biomarkers of tobacco exposure:**<br>Anabasine, cotinine<br>**Biomarkers of cocaïne exposure:**<br>Benzoylecgonine, cocaine, ecgonine methyl ester<br>**Pharmaceuticals:**<br>Amoxicillin
**14***	**Biomarkers of tobacco exposure:**<br>Anabasine<br>**Biomarkers of cannabis exposure:**<br>11-OH-THC, THC-COOH-glucuronide, THC-COOH, cannabicitran, cannabinodiol, THC<br>**Pharmaceuticals:**<br>Bromazepam, 3-hydroxybromazepam, lorazepam, lormetazepam, midazolam, hydroxymidazolam, nordiazepam, oxazepam, temazepam, cefotaxime, domperidone, laudanosine, morphine, N-desmethyltramadol, O- desmethyltramadol, tramadol
**15**	**Biomarkers of tobacco exposure:**<br>Anabasine<br>**Pharmaceuticals:**<br>Darunavir, lamivudine, lidocaine, nevirapine, norfentanyl, norlidocaine, ropivacaine, zidovudine
**16**	**Biomarkers of tobacco exposure:**<br>Cotinine, anabasine<br>**Biomarkers of cannabis exposure:**<br>THC-COOH-glucuronide, cannabinol<br>**Pharmaceuticals:**<br>3-hydroxylidocaine, lidocaine, norlidocaine, ropicavaine, methylphenidate, norfentanyl
**17**	**Biomarkers of tobacco exposure:**<br>Anabasine, cotinine<br>**Pharmaceuticals:**<br>Lidocaine, norfentanyl, norlidocaine, paracetamol, ropivacaine
**18**	**Biomarkers of tobacco exposure:**<br>Anabasine, cotinine<br>**Pharmaceuticals:**<br>EDDP, methadone, midazolam, hydroxymidazolam, levetiracetam, morphine, paracetamol
**19**	**Biomarkers of tobacco exposure:**<br>Anabasine<br>**Pharmaceuticals:**<br>Amoxicillin, codeine, cotinine, lamotrigine, morphine, morphine-3-glucuronide, norcodeine, normorphine, paracetamol, nordiazepam, oxazepam, temazepam, zolpidem
**20**	**Biomarkers of tobacco exposure:**<br>Anabasine, cotinine<br>**Pharmaceuticals:**<br>Desmethylnefopam, norfentanyl, tramadol

**21**	**Biomarkers of tobacco exposure:**<br>Anabasine, cotinine<br>**Biomarkers of cannabis exposure:**<br>THC-COOH<br>**Pharmaceuticals:**<br>3-hydroxyropivacaine, lidocaine, norfentanyl, norlidocaine, ropivacaine
**22**	**Biomarkers of tobacco exposure:**<br>Anabasine, cotinine<br>**Biomarkers of cannabis exposure:**<br>Cannabinol, THC-COOH, THC<br>**Pharmaceuticals:**<br>Ropivacaine, 3-hydroxyropivacaine, lidocaine, norlidocaine, 3-hydroxylidocaine, amoxicillin, cefazolin, ephedrine, lamotrigine, norfentanyl, venlafaxine, N-desmethylvenlafaxine, O-desmethylvenlafaxine
**23**	**Biomarkers of tobacco exposure:**<br>Anabasine, cotinine<br>**Pharmaceuticals:**<br>Lidocaine, 3-hydroxylidocaine, ropivacaine, hydroxyzine, cetirizine, 1-(4-chlorophenyl) -phenylmethyl piperazine, codeine, morphine, N-desmethyltramadol, norfentanyl, paracetamol
**24**	**Biomarkers of cannabis exposure:**<br>THC-OH, THC-COOH<br>**Pharmaceuticals:**<br>Lidocaine, norlidocaine, norfentanyl, ropivacaine
**25**	**Biomarkers of tobacco exposure:**<br>Anabasine, cotinine<br>**Pharmaceuticals:**<br>Hydroxyzine, 1-(4-chlorophenyl) -phenylmethyl piperazine, bupivacaine, cimetidine, labetalol, lormetazepam, nicardipine, nordiazepam, oxazepam, temazepam, paracetamol, sertraline, zolpidem
**26**	**Biomarkers of tobacco exposure:**<br>Anabasine, cotinine<br>**Biomarkers of cannabis exposure:**<br>Cannabinol, THC-COOH, THC, 11-OH-THC<br>**Pharmaceuticals:**<br>Lidocaine, norlidocaine, ropivacaine
**27**	**Pharmaceuticals:**<br>7-aminoclonazepam, lamotrigine, levetiracetam, norfentanyl, norlidocaine, paracetamol
**28**	**Pharmaceuticals:**<br>Amoxapine, loxapine, clonidine, oxazepam
**29**	**Biomarkers of tobacco exposure:**<br>Anabasine<br>**Pharmaceuticals:**<br>Lidocaine, metoclopramide, morphine, norlidocaine, tramadol
